# Updated Title

**DOI:** 10.1001/jamanetworkopen.2024.6717

**Published:** 2024-03-15

The title of the Invited Commentary “Prevalence of Children Who Only Consume Breast Milk Across 92 Low- and Middle-Income Countries,”^1^ published February 12, 2024, was updated to reflect the definition of “zero-food children” in the Original Investigation “Prevalence of Children Aged 6 to 23 Months Who Did Not Consume Animal Milk, Formula, or Solid or Semisolid Food During the Last 24 Hours Across Low- and Middle-Income Countries” by Karlsson et al.^2^ The updated title reads, “Prevalence of Inadequate Nutrition Among Children Aged 6 to 23 Months in Low- and Middle-Income Countries.”^1^
